# Simultaneous Clamping and Cutting Force Measurements with Built-In Sensors

**DOI:** 10.3390/s20133736

**Published:** 2020-07-03

**Authors:** Sina Rezvani, Chang-Ju Kim, Simon S. Park, Jihyun Lee

**Affiliations:** 1 Mechanical and Manufacturing Engineering, University of Calgary, 2500 University Drive NW, Calgary, AB T2N 1N4, Canada; sina.rezvani@ucalgary.ca (S.R.); simon.park@ucalgary.ca (S.S.P.); 2 Ultra-Precision Machines and Systems, Korea Institute of Machinery and Materials, 156 Gajeongbuk-Ro Yuseong-Gu, Daejeon 34103, Korea; changjukim@kimm.re.kr

**Keywords:** clamping forces, cutting forces, vise with built-in sensors, PZT fabrication, sensor fusion

## Abstract

The intensity of the clamping force during milling operations is very important, because an excessive clamping force can distort the workpiece, while inadequate clamping causes slippage of the workpiece. Since the overall clamping force can be affected by the cutting forces throughout machining, it is necessary to monitor the change of clamping and the cutting forces during the process. This paper proposes a hybrid system in the form of a vise with built-in strain gauges and in-house-developed piezoelectric sensors for simultaneous measurement of clamping and cutting forces. Lead zirconate titanate (PZT) sensors are fabricated and embedded in a layered jaw to measure the dynamic forces of the machine tool. A cross-shaped groove within the jaw is designed to embed strain gauges, which predominantly measure the static clamping forces. Sensor fusion technology combining the signals of the strain gauges and PZT piezoelectric sensors is used to investigate the interactions between cutting forces and clamping forces. The results show average errors of 11%, 17%, and 6% for milling forces in X, Y, and Z directions, respectively; and 19% error for clamping forces, confirming the capability of the setup to monitor the forces in milling.

## 1. Introduction

Global competition has posed a major challenge to industry, especially the manufacturing sector. While production cost and time are paramount, there are other expectations regarding the accuracy and quality of the products. Each production stage should be monitored constantly to obtain optimum operating conditions. One of the preliminary steps for precision manufacturing is to ensure that the workpiece is held stationary during the machining process. The efficiency of a workholding system can be evaluated by its capability to achieve workpiece stability, positioning accuracy, minimum workpiece displacement and distortion, and minimum interference with the cutting tool [1].

Machining vises are the most common devices used to hold a workpiece securely during different machining operations, such as milling and drilling. The vises consist of the main body, two jaws (one fixed and one adjustable), along with a handle and a screw used to move the adjustable jaw and apply the clamping force. The workpiece must be stable within the vise throughout the machining process, which means the vise should fully restrain the workpiece subjected to static clamping forces and dynamically varying machining forces. Otherwise, either lift-off or macro-slippage occurs at the jaw–workpiece contacts during the machining process [2]. The clamping forces can also distort the workpiece. The distortion will cause dimensional inaccuracy if the clamping force is unnecessarily high [3]. Consequently, measuring the intensity of the clamping force while machining is very important to prevent the workpiece’s slippage or deformation.

In milling operations, the most important parameter that affects the static clamping force is the dynamically varying cutting forces. Clamping forces are typically measured separately from the cutting forces using hydraulic gauges. Horie [4] developed a machine vise that included a strain detector device located between the supports and flange of the feed screw to detect the axial static force. Since the magnitude of the cutting forces may loosen the clamping and cause lift-off or micro-slippage of the workpiece, some studies have investigated the interactions of the cutting and clamping forces and their effects on the machining quality. Although the majority of these studies are limited to theoretical and finite element (FE) models [5,6,7,8], several setups have also been developed to experimentally investigate the effects of dynamic forces on the clamping force [9,10]. Sun et al. [9] introduced a sensing system with embedded polyvinylidene fluoride (PVDF) thin-film sensors to monitor the dynamic clamping force in milling operations of thin-walled structures. Gupta [10] developed a drilling fixture consisting of a standard vise and two dynamometers to find the effects of thrust force and torque on the minimum required clamping force during drilling. The vise was mounted on a dynamometer to measure the thrust force and torque, while the clamping forces were measured using the other dynamometer attached to the fixed jaw of the vise. A linear increase of the minimum required clamping force with the increase of thrust force was reported. Overall, developing a new setup with the capability of measuring the clamping and cutting forces seems necessary to investigate the effects of the dynamic machining forces on the applied clamping force.

To monitor and control the machining operations, it is also crucial to measure the cutting forces accurately. Cutting forces are used to understand the cutting mechanism and the process of chip formation [11], optimize the manufacturing parameters [12], and monitor real-time tool wear and breakage [13,14,15]. Cutting force measurements are also necessary to detect chatter vibration and workpiece stability [16]. Moreover, the cutting forces are the most important indicators of machining status and quality [17]. Because of the importance of cutting forces, various devices have been developed to enhance the force measurement in terms of resolution, accuracy, and frequency bandwidth. Teti et al. [18] have undertaken a detailed review of sensors used to monitor the machining operations. The methods used to measure the cutting forces can be categorized into two major groups—indirect and direct measurements. In the former method, the cutting forces are estimated using the data gathered from different types of sensors, such as accelerometers [19], sensors that measure the current of spindle and feed motors [20,21], and non-contact displacement-based sensors (e.g., capacitive sensors [22,23] and optical fibers [24]). Although these techniques are usually more cost-effective, they have some limitations related to the frequency bandwidth and resolution. The latter method refers to the direct measurement of cutting forces using different types of force sensors, such as piezoelectric sensors [25,26] and strain gauges [27,28]. The key advantages of piezoelectric sensors compared with other force sensors are their robustness, small size, and self-generated signals [28]. Due to the increased rigidity and improved dynamic range, piezoelectric dynamometers are more popular than strain gauge-based sensors [28]. However, the electric signals generated by the piezoelectric crystal sensors decays over time, which makes them unsuitable for measuring static forces.

This paper proposes a new force-sensing device with the capability of measuring cutting and clamping forces simultaneously. The device allows experimental investigation of interactions between cutting and clamping forces during the milling operations. Lead zirconate titanate (PZT) sensors are used to measure dynamic forces. PZT elements with different thicknesses are fabricated to investigate the sufficient thickness of the sensors for the setup. Afterwards, PZT elements with the selected thickness are poled in shear and axial directions, and their piezoelectric coefficients, modulus of elasticity, and hardness are measured. This paper also proposes the use of strain gauges for the measurement of the static forces because the electrostatic charge of PZT piezoelectric sensors decreases over time. A jaw with a newly designed cross-shaped groove is used to maximize the strains. Fusing signals of the piezoelectric sensors and strain gauges is proposed, such that the clamping and cutting forces can be monitored simultaneously during the milling process without sacrificing the frequency bandwidth. The outline of this paper is as follows: In Section 2, the manufacturing process for the PZT sensors and the design of the developed sensing system are described. In Section 3, the experimental setups and the calibration methods are explained. Section 4 presents the results of clamping and cutting force measurements and illustrates the method used to fuse the signals. Finally, Section 5 describes the conclusions.

## 2. Design and Fabrication of Force Sensing System

### 2.1. Fabrication of PZT Piezoelectric Sensors

The great properties of piezoceramics, including their robustness and low manufacturing cost, have increased their potential applications for sensing and actuation compared with other piezoelectric materials. PZT is presently the most widely used piezoceramic because of its great piezoelectric coefficients and high operating temperatures [29]. Since PZT is a ferroelectric ceramic, it possesses higher piezoelectric coefficients compared to natural materials, such as quartz [29]. However, piezoelectric ceramics are usually too brittle and can crack easily during assembly and testing. Thicker piezoelectric elements can be used to increase the robustness of the element, but very thick sensors may potentially have an adverse impact on the dynamics of the structure and decrease the bandwidth of the system. A compromise is needed between the structural robustness and the dynamic response.

This paper proposes the fabrication of highly sensitive shear and axial sensors of sufficient thickness. The width and length of the PZT elements were chosen based on the dimensional limitations of the design. Piezoelectric elements with different thicknesses ranging from 1 to 2.5 mm were fabricated and clamped in a layered jaw to find the minimum required thickness for this application. Since it was observed that elements with a thickness of less than 2 mm break easily when static load is applied, a thickness of 2.5 mm was chosen for the PZT elements. To manufacture the piezoelectric sensors, lead zirconate titanate Navy Type VI (PZT-5H) powder was used, with an average size of less than 600 nm and a Curie temperature of 130 °C (TCERA CO.). The powder included some sintering aid additives (nickel and niobium) to decrease the sintering temperature from 1250 to 950 °C. Based on the datasheet provided by the manufacturer, this material, which is a soft PZT, has a high piezoelectric coefficient ($d_{33}$ of 600–700 picocoulombs per newton (pC/N)), high electromechanical coupling factor (planar electromechanical coupling, $k_{p}\approx 0.64$), and high relative dielectric constant ($K^{T}\approx 3800$). The chemical composition of the powder is listed in Table 1.

Polyvinyl alcohol (PVA) is used as a temporary binder, which helps to preserve the shape of the green bodies after pressing. This is done by covering the surface of the particles and making them stick together via the interlocking chains during the pressing process [30]. In this research, PVA with an average molecular weight of 61,000 (MilliporeSigma Canada Co., Etobicoke, Canada) was used as the binder.

**Figure 1:**
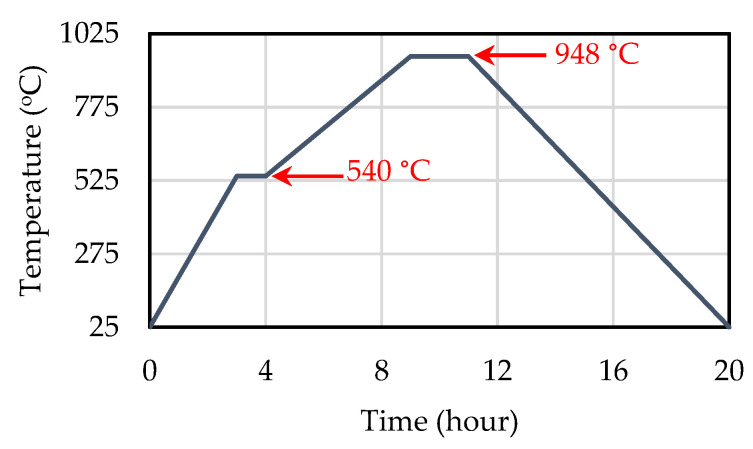
Temperature–time program used to sinter the PZT plates.

To fabricate the sensors, first 5 g PVA was dissolved in 100 mL DI water using magnetic stirring at 80 °C. Then, 5 mL of the resultant solution and 50 g PZT powder were homogeneously mixed using a mortar and pestle, and the particles were ground and dried at 120 °C for 20 min to achieve a finer powder. Afterward, 6 g of the fine powder was poured into a die (20 × 20 mm) and pressed at 200 MPa. This optimal pressing pressure was obtained through trial and error to reach a compact density of about 55%, which is sufficient for the effective manufacturing of bulk PZT samples [31]. Sintering was performed in a furnace tube at a specific time–temperature profile provided by the manufacturer, with a maximum temperature of 948 °C, as shown in Figure 1. The first plateau occurring at 540 °C involves combustion of the binder system. Due to the densification during the sintering process, the final size of the piezoelectric elements was 17 × 17 × 2.5 mm.

**Figure 2:**
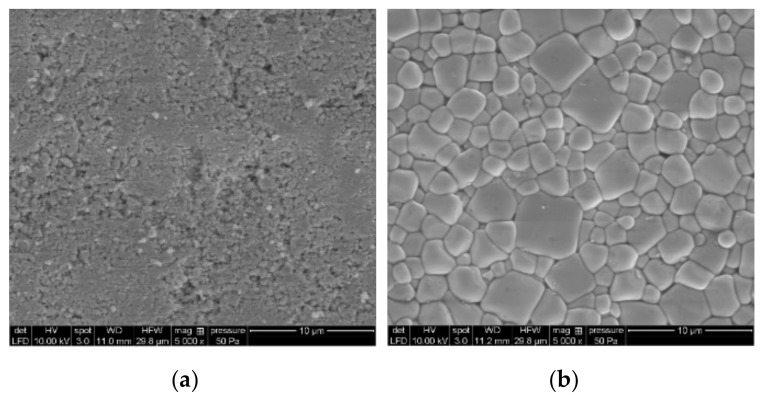
SEM image of the sample (**a**) before and (**b**) after sintering.

Figure 2 shows the SEM images of a sample before and after sintering. The images clearly show successful PZT powder crystallization after sintering. The average size of the grains was 2.24 µm, which was estimated by ImageJ software.

A high voltage poling process was conducted to align the randomly oriented dipoles in the piezoelectric material. Generally, there are two poling methods for piezoelectric materials, including corona poling, which is mostly used for polymer-based piezoelectric materials, and contact poling, in which the electric field is applied directly to the element through the electrodes printed on two sides of it. Due to the possibility of using a lower electric voltage, the latter method is more efficient and is normally used to pole piezoceramics. The samples were poled through their thickness to make axial sensors. The sintered PZT plates were brushed with silver paint on both sides and placed in the contact poling setup. The poling setup was immersed in silicone oil. A DC electric field of 2.5 kV/mm was applied and the temperature increased to 125 °C, which is just below the Curie temperature of the PZT powder. The elevated temperature helps with the alignment of the dipoles in the material and results in higher piezoelectric coefficients. The temperature was kept at 125 °C for 2 h and then allowed to cool to room temperature, after which the electric field was removed.

**Figure 3:**
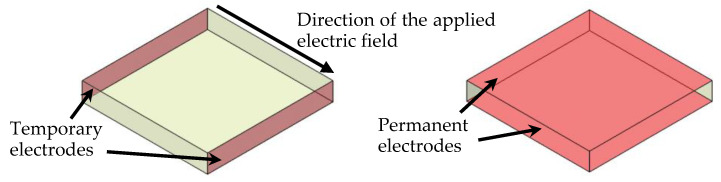
Temporary and permanent electrodes for the shear sensors.

**Figure 4:**
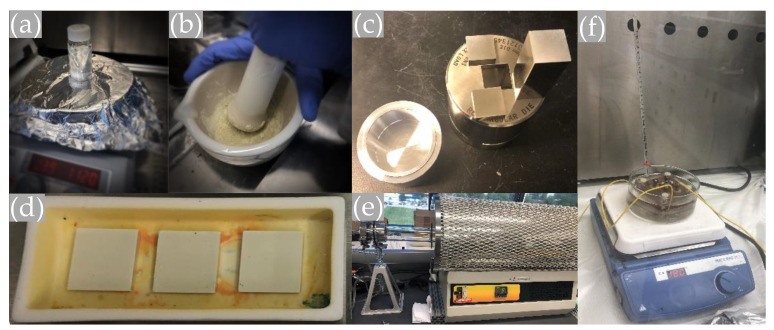
The fabrication procedure for the piezosensors: (**a**) dissolving polyvinyl alcohol (PVA) in deionized (DI) water; (**b**) mixing and grinding PZT powder with the PVA solution; (**c**) the mold used to press the powder; (**d**) pressed samples placed in the alumina boat; (**e**) furnace tube used to sinter the powder; and (**f**) the poling process under controlled voltage and temperature.

Although the poling process was the same for the shear sensors, the poling direction was different, as shown in Figure 3. For the shear sensors, temporary electrodes were used in the direction perpendicular to the permanent electrodes. After poling, the temporary electrodes were removed, and the permanent ones were brushed with silver paint. Figure 4 provides an illustration of the sensor fabrication procedure in detail.

The piezoelectric strain coefficient is the ratio of the electric displacement to the applied stress (at zero electric field) or the ratio of the strain to the applied electric field (at zero stress). When both the applied force and generated charges are in the poling direction, the axial piezoelectric coefficient ($d_{33}$) can also be defined as the ratio of electric charges (Q) linearly generated in response to the applied force (F) [32]:

(1) $$d_{33}=\frac{Q}{F}$$

**Figure 5:**
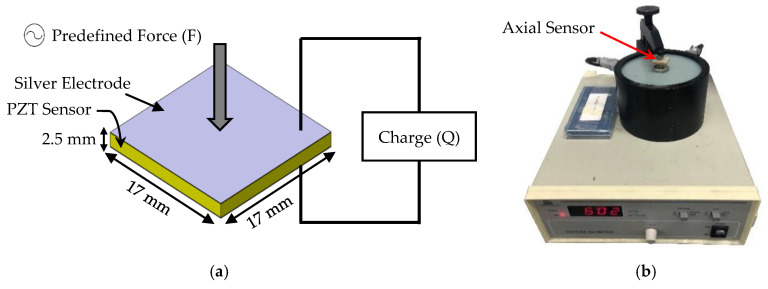
(**a**) The technique used to measure the piezoelectric coefficient ($d_{33}$). (**b**) Measurement of the piezoelectric coefficient ($d_{33}$) of the axial sensors.

Figure 5a shows the technique used to measure the $d_{33}$ coefficient. The piezoelectric coefficient of the axial sensors was measured using a wide range $d_{33}$ tester (APC YE2730A $d_{33}$ meter), as shown in Figure 5b. The $d_{33}$ of the fabricated samples is about 600 pC/N, which is comparable to the $d_{33}$ of commercial Navy Type VI elements (630 pC/N) [29].

The shear piezoelectric coefficient ($d_{15}$) can be defined as the ratio of electric charges (Q) linearly generated in response to the applied shear force (V):

(2) $$d_{15}=\frac{Q}{V}$$

**Figure 6:**
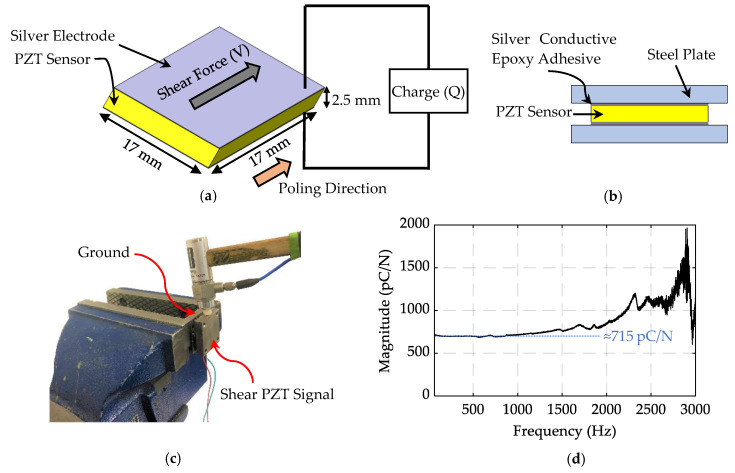
(**a**) Direction of applied shear force with respect to the poling direction to measure $d_{15}$ (**b**) Schematic of the developed setup to measure $d_{15}$. (**c**) Modal hammer test conducted on the clamped setup. (**d**) Frequency response function (FRF) result used to estimate the $d_{15}$ value of the shear elements

Figure 6a illustrates the direction of the applied shear force and the poling direction with respect to the electrodes printed on the shear element. The piezoelectric coefficient of the shear sensors was measured using modal hammer tests. The shear piezoelectric element was inserted between two steel plates and silver conductive epoxy adhesive was used to attach the steel plates to the piezoelectric element, as shown in Figure 6b. One of the plates was clamped on a vise and the force was applied to the other plate in the poling direction by the modal hammer (PCB 208A03), as illustrated in Figure 6c. The applied force of the hammer and the generated electrostatic charge on the piezoelectric element were measured using a charge amplifier (Kistler 5010) and a data acquisition instrument (NI 9234 DAQ). Figure 6d shows the average frequency response function (FRF) of the input force and the output charge for the five tests. The magnitude of the initial flat area on the curve shows the generated electric charges due to the applied force of 1 N; in other words, it shows the piezoelectric coefficient of the sensor in pC/N. Based on the results, the $d_{15}$ of the fabricated samples is about 715 pC/N, which is similar to the $d_{15}$ value of commercial Navy Type VI elements (720 pC/N) [29].

**Figure 7:**
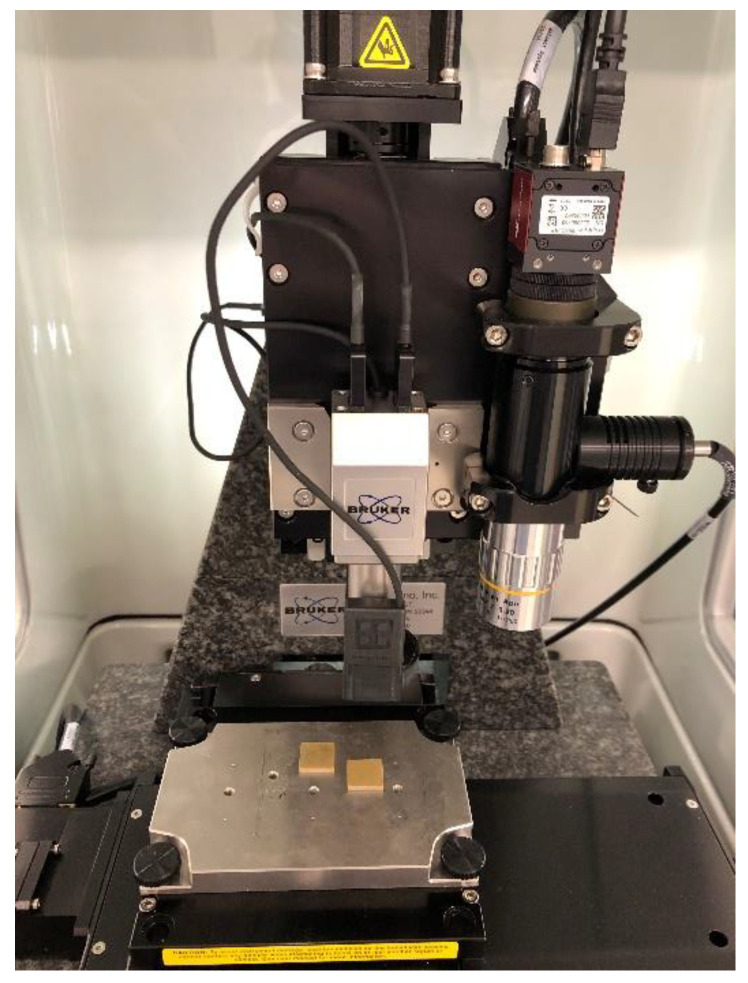
Measuring the hardness and modulus of elasticity of samples using a micro-indenter device.

The modulus of elasticity and hardness of the sintered samples were measured by a micro-indenter device (Hysitron TI Premier, Bruker, Beijing, China) with a Berkovich probe, as shown in Figure 7. Equations (3) and (4) can be used to extract these properties [33]. In this method, the slope of the tangent line to the unloading curve (S) for the first increments (start of unloading) is required to calculate the elastic modulus (E), while the hardness (H) is the ratio of the maximum applied load ($P_{max}$) to the projected contact area (A).

(3) $$E=\frac{1-\nu^{2}}{\frac{2\beta \sqrt{A}}{S\sqrt{\pi }}-\frac{1-\nu_{i}^{2}}{E_{i}}}$$

(4) $$H=\frac{P_{max}}{A}$$

where ν is Poisson’s ratio of the PZT ceramic (0.34) [34], $\nu_{i}$ is Poisson’s ratio of the diamond indenter (0.07) [35], $E_{i}$ is the modulus of elasticity of the diamond indenter (1141 GPa) [35], and β is 1.034 for the Berkovich probe [36].

The indentation tests were conducted at 16 points on the surface of the sample and the average values were reported. The results showed that the modulus of elasticity of the sample was 52 GPa, which is almost the same as the ones reported for commercial Navy Type VI elements (51 GPa) [29], and the hardness of the sample was 4.1 GPa, which is comparable to the hardness reported by Jiansirisomboon et al. [37] for pure PZT and lead magnesium niobite-lead zirconate titanate (PMN-PZT) ceramics (3.80-5.64 GPa).

### 2.2. Development of Vise with Built-In Sensors

**Figure 8:**
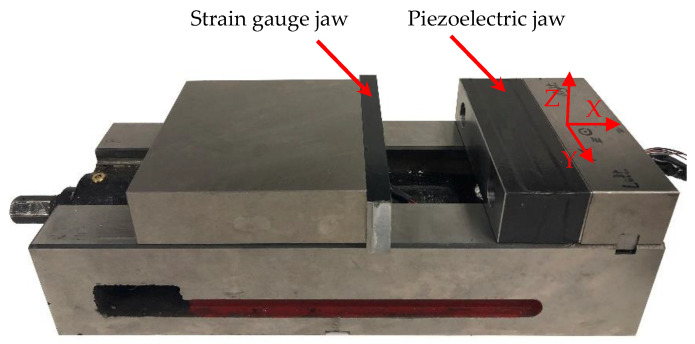
Jaws with embedded strain gauges and piezoelectric sensors.

A new vise setup with two different jaws was proposed to measure clamping and cutting forces simultaneously. One jaw located at the movable end of the vise includes strain gauges with pre-attached cables (Vishay Electric C4A-06-060SL-350-39P) to measure the static force. The strain gauges have a resistance of 350 Ω, gauge factor of 1.5, strain range of ±3%, operating temperature of between –51 and 80 °C, and a linear pattern with an active length and width of 1.52 mm and 2.54 mm, respectively. The other jaw located at the fixed end of the vise embeds the PZT piezoelectric sensors to measure the dynamic forces. This jaw is layered to embed six piezoelectric sensors for X, Y, and Z directions. One layer includes two axial piezoelectric sensors to measure the *X*-axis cutting forces, and two layers include four shear piezoelectric sensors (two per each layer) to measure *Y*-axis and *Z*-axis forces. All jaws are fastened to the rest of the vise assembly through standard bolts, which are generally used to attach the normal jaws to the vise. Thus, the setup can be easily mounted on every vise with the same screw patterns, and the only modification needed is replacing the normal jaws with the newly designed ones. Figure 8 shows the assembled vise with jaws, including strain gauges and the PZT piezoelectric sensors.

**Figure 9:**
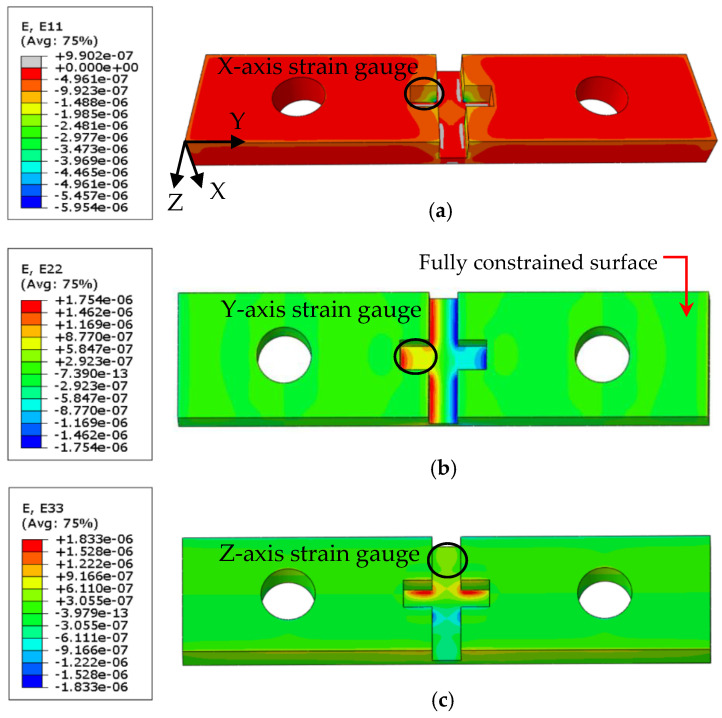
Simulation results for (**a**) *X*-axis strain results from the force in the X direction, (**b**) *Y*-axis strain results from the force in the Y direction, and (**c**) *Z*-axis strain results from the force in the Z direction.

**Figure 10:**
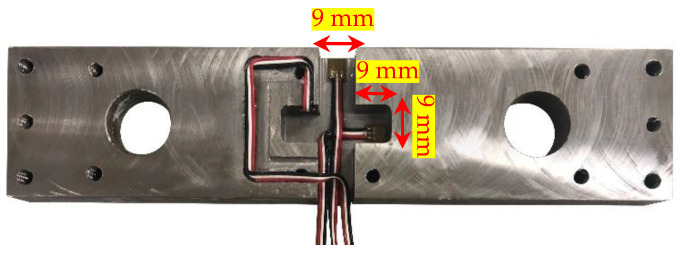
Cross-shaped groove design for integrating strain gauges.

Strain gauges should be attached to the locations with maximum strain in the desired direction and minimum crosstalk in the other directions to measure the static forces accurately. Moreover, the jaw design should result in high strain, while maintaining sufficient stiffness. To satisfy all these conditions, a cross-shaped groove in the center section of the jaw was designed, machined, and used to attach the strain gauges. The locations of strain gauges were selected using a model in finite element (ABAQUS/CAE^TM^ 2019) software. Eight-node linear brick elements with reduced integration (C3D8R) and a maximum size of 0.7 mm were used for the simulations. Distributed static loads with resultant values of 0.5 kN were applied separately in X, Y, and Z directions to the front surface, while the back surface of the jaw was fully constrained. The resultant strains were extracted and averaged on the potential areas for X, Y, and Z strain gauges separately, as shown in Figure 9. The locations with the maximum desired strains and the minimum crosstalk were chosen to attach the strain gauges, as shown in Figure 10.

Table 2 summarizes the crosstalk results from the simulations. The results show that the forces in Y and Z directions have an insignificant effect on the *X*-axis strain gauge, which is utilized to measure the clamping force. Note that the effect of the force in the *X*-axis direction is noticeable on the *Y*-axis strain. The crosstalk can be compensated using the inverse matrix. The relationship between the forces measured by the strain gauge system ($F_{mx}$, $F_{my}$ and $F_{mz}$) and the actual forces ($F_{ax}$, $F_{ay}$ and $F_{az}$) applied to the strain jaw is defined using the following equation:

(5) $$\left\{\begin{array}{c} F_{mx}\\ F_{my}\\ F_{mz} \end{array}\right\}=\left[\begin{array}{ccc} \propto_{xx} & \propto_{xy} & \propto_{xz}\\ \propto_{yx} & \propto_{yy} & \propto_{yz}\\ \propto_{zx} & \propto_{zy} & \propto_{zz} \end{array}\right]\left\{\begin{array}{c} F_{ax}\\ F_{ay}\\ F_{az} \end{array}\right\}=\left[\begin{array}{ccc} 1 & -0.23 & 0.21\\ 0.41 & 1 & 0\\ -0.17 & 0 & 1 \end{array}\right]\left\{\begin{array}{c} F_{ax}\\ F_{ay}\\ F_{az} \end{array}\right\}$$

where $\propto_{ij}$ is the crosstalk, and i and j take the values of *x*, *y*, and *z*. With the inverse matrix, the actual forces can be calculated using each set of simultaneously measured data from the strain gauges. The equation below shows how the actual forces can be reconstructed using the measured values:

(6) $$\left\{\begin{array}{c} F_{ax}\\ F_{ay}\\ F_{az} \end{array}\right\}={\left[\begin{array}{ccc} \propto_{xx} & \propto_{xy} & \propto_{xz}\\ \propto_{yx} & \propto_{yy} & \propto_{yz}\\ \propto_{zx} & \propto_{zy} & \propto_{zz} \end{array}\right]}^{-1}\left\{\begin{array}{c} F_{mx}\\ F_{my}\\ F_{mz} \end{array}\right\}=\left[\begin{array}{ccc} 0.88 & 0.20 & -0.19\\ -0.36 & 0.92 & 0.08\\ 0.15 & 0.03 & 0.97 \end{array}\right]\left\{\begin{array}{c} F_{mx}\\ F_{my}\\ F_{mz} \end{array}\right\}$$

**Figure 11:**
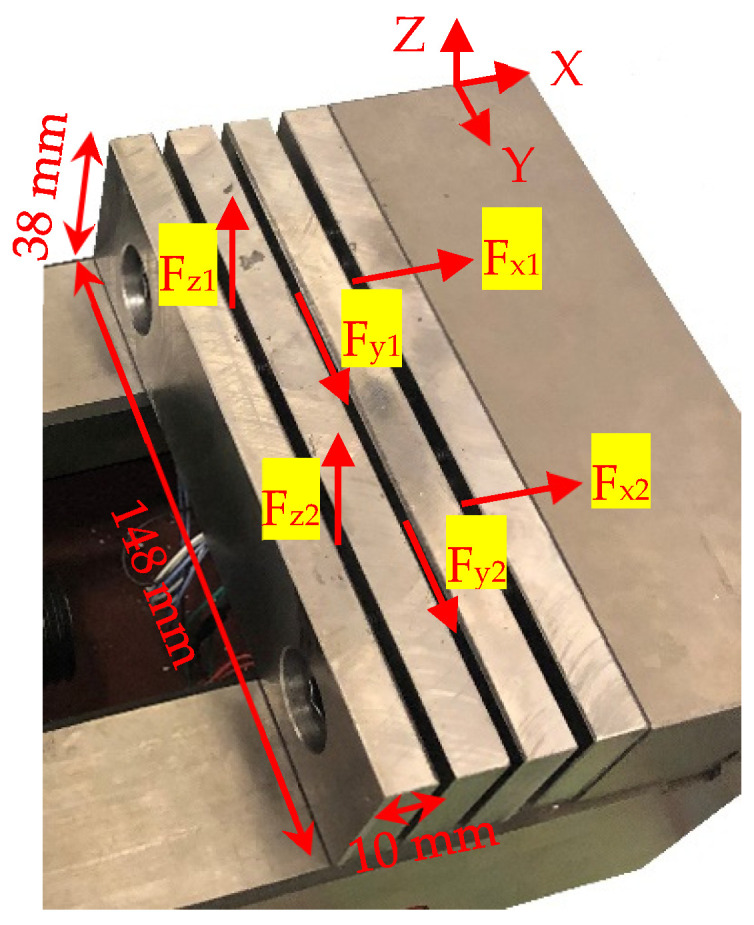
Jaw design with built-in piezoelectric sensors.

The PZT piezoelectric sensors were embedded on the layered jaw located at the fixed end of the vise to measure the dynamic part of the forces. Two shear piezoelectric sensors were embedded between the first and second layers to measure the force in the Z direction. The force in the Y direction was measured using two shear sensors inserted between the second and third layers. The axial piezoelectric sensors were embedded between the third and fourth layers to measure the *X*-axis force. An even preload was applied to each piezoelectric element using a torque of 0.25 N·m to tighten the eight screws between every two layers of the jaw. Both shear and axial piezoelectric sensors had the same dimensions, 17 × 17 × 2.5 mm. Flexible copper-cladded polyimide laminates were used as the electrodes on both sides of the piezoelectric sensors sandwiched between them. Figure 11 shows the design and dimensions of the layered jaw, which was manufactured using layers of mild steel. A thickness of 10 mm was chosen to ensure that the layers remained parallel during clamping and machining.

**Figure 12:**
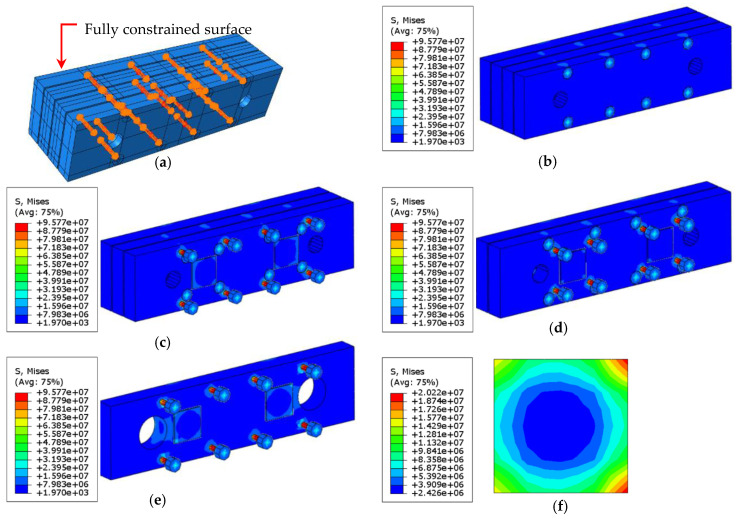
Simulation to investigate the stress field of the piezoelectric sensors and the setup by applying equivalent bolt loads to each screw: (**a**) the boundary conditions and the loads applied on the screws; the stress distribution (Pa) on (**b**) the first layer, (**c**) the second layer and *Z*-axis sensors, (**d**) the third layer and *Y*-axis sensors, (**e**) the fourth layer and *X*-axis sensors, and (**f**) one of the *X*-axis sensors.

It is important to apply a symmetric preload to the piezoelectric sensors; otherwise, they will break easily. In this design, which was validated by finite element simulation, four screws are used to apply a symmetrical preload to each sensor. The equivalent clamping force of the bolt (F) for the applied torque (T) can be estimated using the following equation [38]:

(7) $$F=\frac{T}{KD}$$

where D is the major diameter of the bolt (3.5 mm for the bolt, with a 6-32 UNC thread used in this paper) and K is the coefficient of friction constant, which is 0.2 for steel threads [38]. The FE model was used to evaluate stress. Bolt loads calculated from Equation (7) were applied to the screws, while all six degrees of freedom (DOFs) of the back surface of the jaw were fully constrained. In this simulation, eight-node linear brick elements with reduced integration (C3D8R) and a maximum size of 1 mm were used. As illustrated in Figure 12, the results show that the stress on the piezoelectric sensor is well distributed and symmetrical.

## 3. Force Sensing System Analysis and Experimental Setup

### 3.1. Force Sensing System Analysis

**Figure 13:**
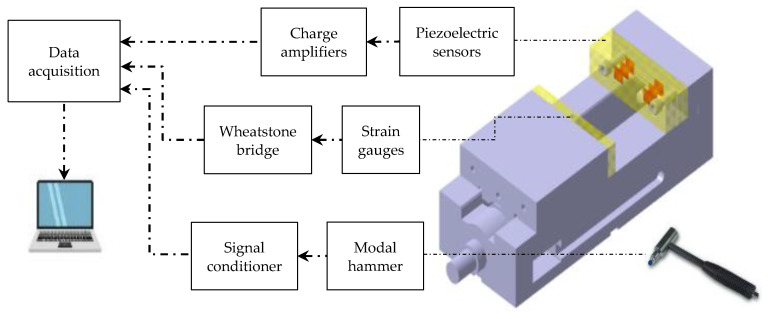
Schematic of calibration tests.

The dynamic behavior of the assembled vise and sensitivities of its sensors need to be investigated to evaluate the bandwidth of the structure and performance of the sensors. The impact hammer tests were performed to find the FRFs of the assembled vise and the sensitivities of the PZT piezoelectric sensors. An impact hammer (PCB 208A03), a data acquisition system (NI 9234 DAQ), and charge amplifiers (Kistler 5010) were used in the impact hammer tests. An electric charge is generated when the hammer hits the workpiece. Charge amplifiers were used to convert the charge to an electric voltage and amplify the signal. Based on the amount of generated charge, which depends on the applied force and the sensitivity of the piezoelectric sensor, an appropriate capacitor should be selected for the charge amplifiers. The signal is finally transmitted through the data acquisition system to a computer for processing. Figure 13 shows the schematic of the calibration tests.

**Figure 14:**
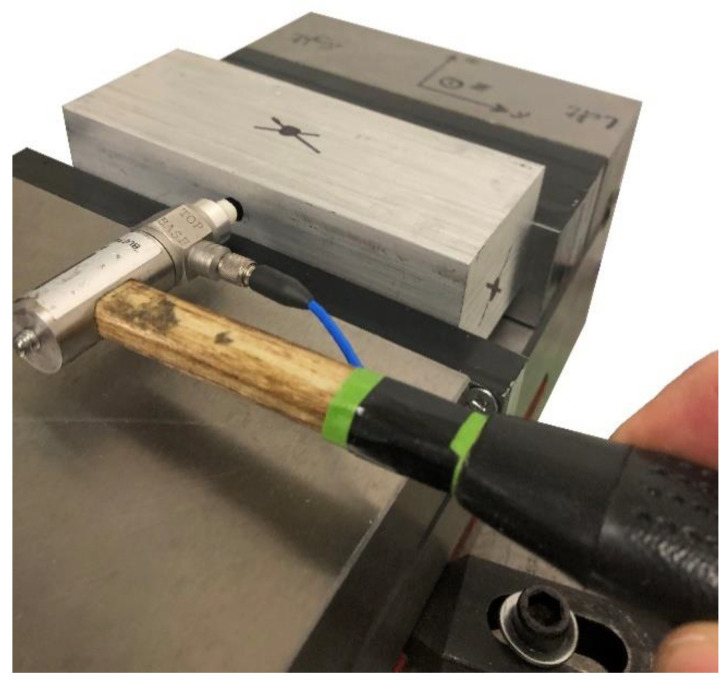
Impact hammer test to find the FRFs.

The entire vise mounted on the milling machine was hit using the impulse impact hammer to measure the FRFs and the sensitivities of the PZT piezoelectric sensors. An accelerometer (PCB 352B10) was used as a reference to compare the results of the piezoelectric sensors. The forces were applied along the center of gravity of the workpiece in X, Y, and Z directions, as shown in Figure 14, and the signals of the sensors were captured. When the signals of hammer and the PZT sensors in the direction of the applied force were captured simultaneously, they were transformed to the frequency domain using discrete fast Fourier transformation (FFT). The FRFs were obtained by dividing the signals of the PZT sensors by the signal of hammer in the frequency domain [25].

**Figure 15:**
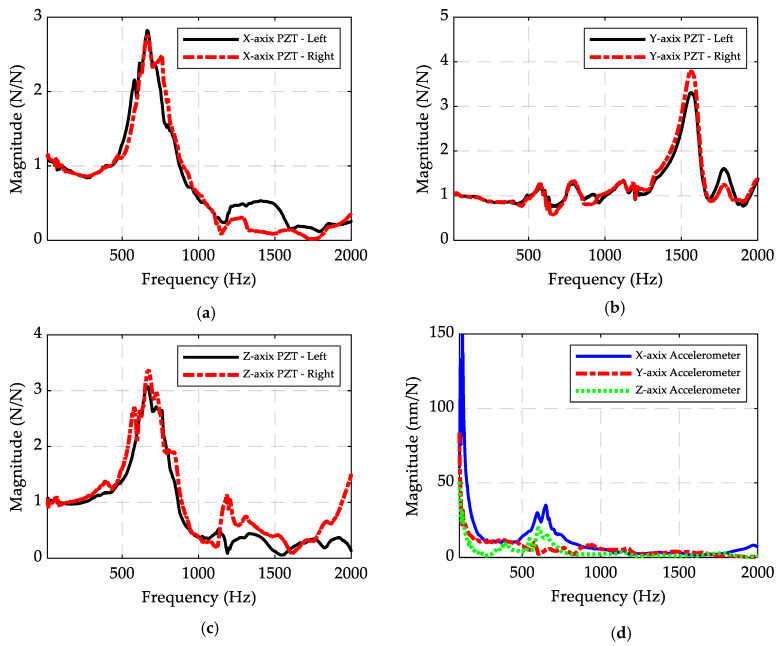
Experimental hammer test FRF results measured by (**a**) piezoelectric sensors in the *X*-axis from the *X*-axis force ($H_{xx}$), (**b**) piezoelectric sensors in the Y-axis from the Y-axis force ($H_{yy}$), and (**c**) piezoelectric sensors in the Z-axis from the Z-axis force ($H_{zz}$); (**d**) and measured by an accelerometer from X-, Y-, and Z-axis forces ($H_{xx}$, $H_{yy}$, $H_{zz}$).

Figure 15 illustrates the FRFs of the axial, *Y*-axis, and *Z*-axis sensors, and the FRFs of the signals captured by the reference accelerometer. The results show that the lowest natural frequency of the system is at about 600 Hz because of the dynamic nature of the workpiece. This frequency is a function of the material and size of the workpiece and the boundary conditions. If a material with a different modulus of elasticity is used or if the workpiece is clamped using a different force, the frequency changes. This frequency restricts the frequency bandwidth of the setup, which is directly related to the sensor performance. The sensitivities of the PZT piezoelectric sensors are depicted in Table 3, which are much higher than the commercial table dynamometers (<30 pC/N), in which quartz piezoelectric elements are normally used [39]. There is a difference between the sensitivity values for the sensors on the right side and left side of the jaw for the *X*-axis and *Z*-axis sensors, which is caused by the difference in the preloads applied to these sensors and different contact resistances between the sensors and electrodes due to the inherent manufacturing challenges.

The *X*-axis strain gauge was calibrated by applying clamping force and comparing the results with a reference force sensor. The strain gauges in Y and Z directions were calibrated by conducting cutting tests and comparing the average DC value with a reference sensor. Strain variations were measured using a Wheatstone bridge (quarter bridge configuration type I). Table 3 describes the sensitivities of strain gauges.

### 3.2. Experimental Setup for Measuring Clamping and Cutting Forces

**Figure 16:**
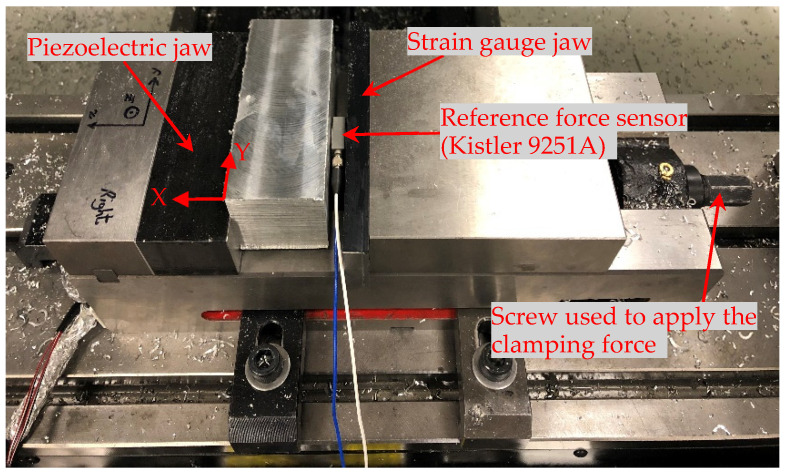
The setup used for the clamping test.

The clamping test was performed based on the setup shown in Figure 16. A three-axis quartz piezoelectric force sensor (Kistler 9251A) was used as a reference sensor, with a sensitivity value and range of 3.88 pC/N and ±5 kN in the X direction; and a sensitivity value and range of 8.08 pC/N and ±2.5 kN in the Y and Z directions, respectively. The workpiece was clamped in the vise and the applied clamping force in the X direction was measured by the axial strain gauge, PZT sensors, and the reference sensor simultaneously.

**Figure 17:**
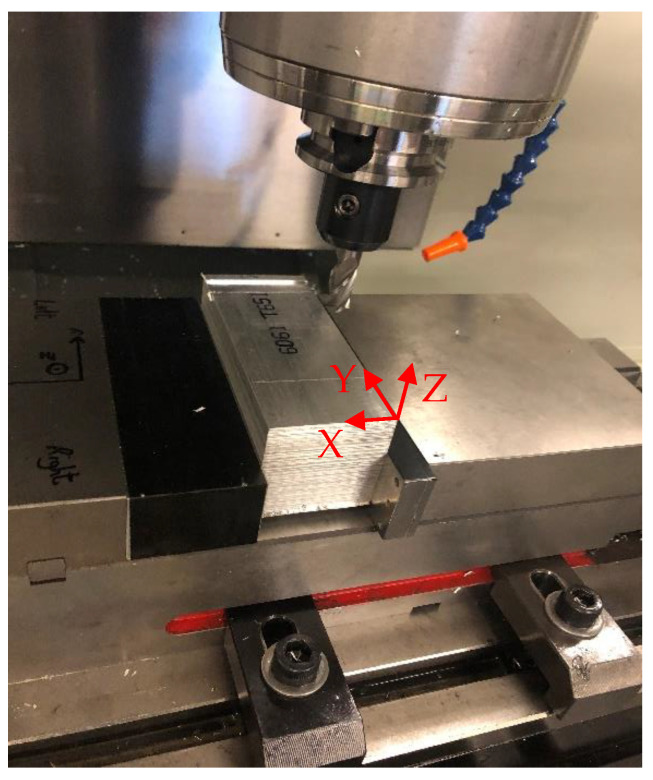
Milling test setup.

Cutting tests were performed using a CNC milling machine (Haas TM-2P). A four-fluted tungsten carbide tool was used for full immersion end milling. The spindle speed of 1500 rpm was selected to investigate the performance of the force-sensing vise. This spindle speed resulted in a tooth pass frequency of 100 Hz. Thus, the tooth passing frequency and its first two harmonics were within the frequency bandwidth of the system and the measured forces were not distorted by the dynamics of the setup. The cutting conditions are summarized in Table 4. Full immersion milling was performed along the *X*-axis of the workpiece, defined in Figure 17, and the cutting forces were calculated by averaging the signals of the right and left sensors in each direction. The reference force sensor was used to validate the performance of the designed vise.

## 4. Experimental Results

### 4.1. Clamping and Cutting Forces

**Figure 18:**
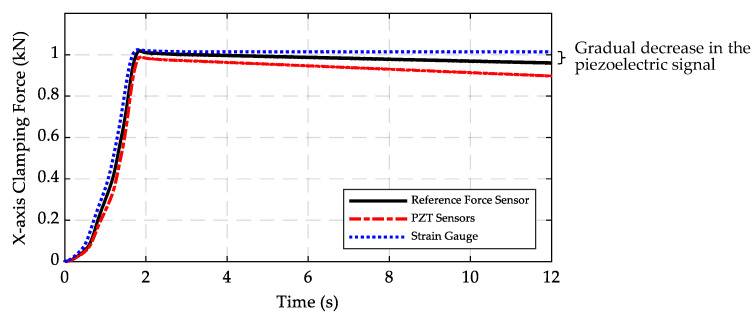
Results of strain gauge and PZT sensors compared with the result for the reference piezoelectric force sensor.

Clamping forces measured by the axial strain gauge, PZT sensors, and the reference force sensor are shown in Figure 18. The measurement errors of the peak value, rise time, and peak time of the strain gauge signal compared with the reference signal are 0.5%, 2.3%, and 1.7%, respectively. Although the PZT sensor signals show only 3% error in measurement of the peak value, there is a gradual decrease in the signal. The results show that after about 10 s, while the strain gauge signal remains almost constant, there is a noticeable decrease (8.7%) in the signal of the PZT sensors due to the leakage of the electric charge on the piezoelectric elements. The signal of the force sensor also decreases (4.9%), albeit less than the PZT sensors, indicating that the circuit of the force sensor has higher insulation compared to the PZT sensors. The gradual decay of the piezoelectric sensors makes them inappropriate for measuring the static forces. Therefore, in this paper, the strain gauges were used to measure the static forces.

**Figure 19:**
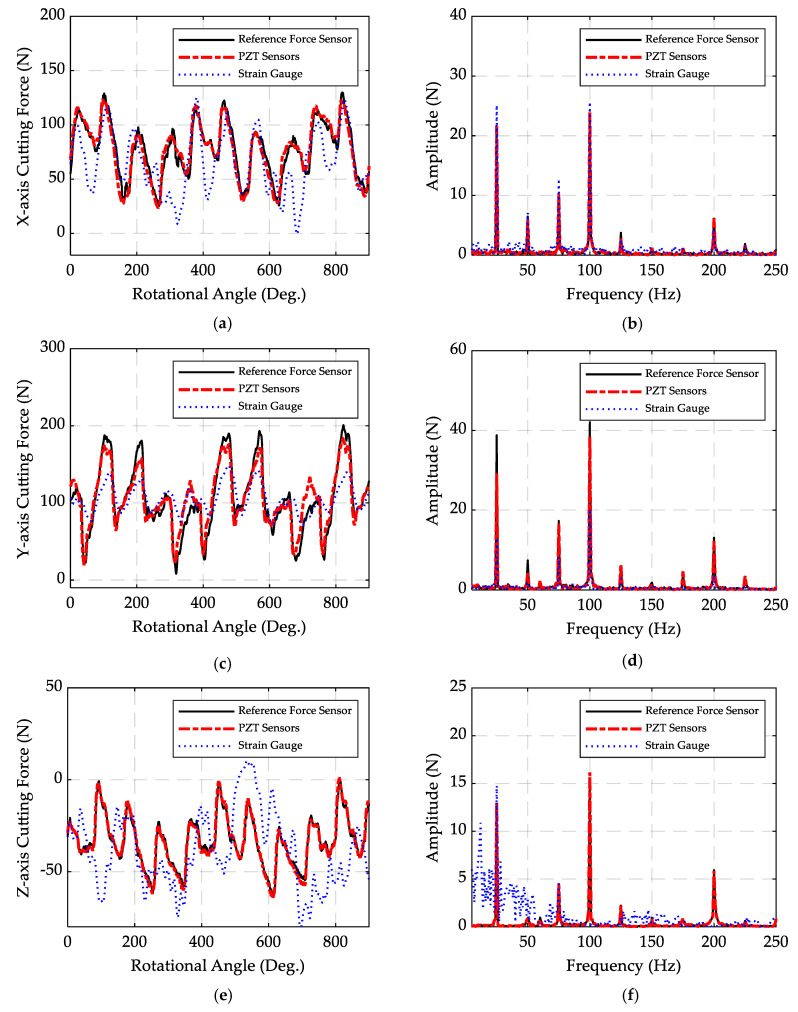
(**a**) Cutting forces measured in the X direction, (**b**) the fast Fourier transform (FFT) of the measured *X*-axis forces, (**c**) cutting forces measured in the Y direction, (**d**) the FFT of the measured *Y*-axis forces, (**e**) cutting forces measured in the Z direction, (**f**) and the FFT of the measured *Z*-axis forces.

The cutting forces were measured by the PZT piezoelectric sensors, strain gauges, and the reference force sensor. Figure 19 shows the measured forces in X, Y, and Z directions, and the FFTs of those forces. The average errors between the signals measured by the PZT sensors and the reference force sensor are 11%, 17%, and 6% for X, Y, and Z directions, respectively, which confirms the reliability of the performance of the PZT sensors. However, the results of the strain gauges include large discrepancies. Figure 19e,f show that the *Z*-axis strain gauge result is not reliable, which is due to the very low sensitivity of the strain gauge system for measuring the force in the Z direction. This can be improved by using a strain gauge with higher sensitivity, although due to the low milling force in the Z direction and the high noise-to-signal ratio for very low strains, it may still be problematic to measure the strain in Z direction accurately. The results prove that while the strain gauge system can measure the static forces more accurately, the results for the PZT elements are more reliable and accurate for cutting force measurement. The FFTs reveal that the dominant frequency in the tests is 100 Hz, which is the tooth passing frequency (considering the spindle speed and number of flutes mentioned in Table 4). Furthermore, there are high peaks at the spindle frequency (25 Hz), which are caused by a slight runout of the cutting tool. The effect of the runout is also obvious in the time domain signals; the magnitudes of the cutting forces are different for each flute.

### 4.2. Fusing Signals from Strain Gauges and PZT Sensors

**Figure 20:**
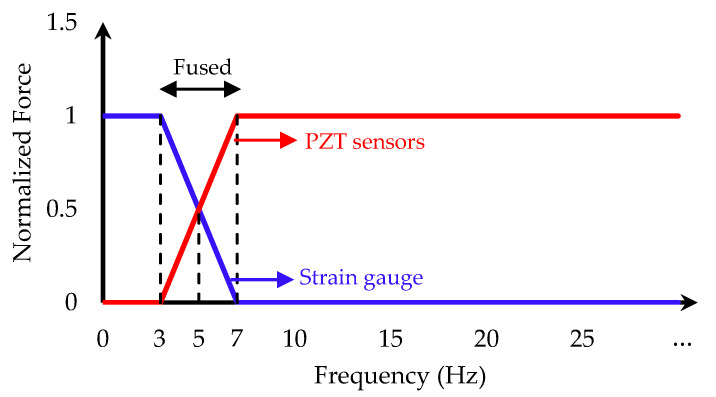
The effects of cutting forces on the clamping force.

The previous section showed that the strain gauge system is more reliable for static and semi-static force measurements, while the PZT piezoelectric sensors can accurately measure the dynamic cutting forces. Thus, the results of the *X*-axis strain gauge and the piezoelectric sensors were fused to provide better prediction of the total clamping force from the start of the workpiece clamping until the end of the milling process. The linear model illustrated in Figure 20 was used to fuse the signals. The forces with frequencies of less than 3 Hz are only measured by the strain gauge system. The results of the strain gauge and the piezoelectric sensors are fused for the frequencies between 3 and 7 Hz, while the weight of the piezoelectric signal increases linearly from zero at 3 Hz to 1 at 7 Hz. The forces with frequencies greater than 7 Hz are only measured by the piezoelectric sensors. With this simple model, strain gauges predominantly measure the DC values, while PZT sensors measure the dynamic forces.

**Figure 21:**
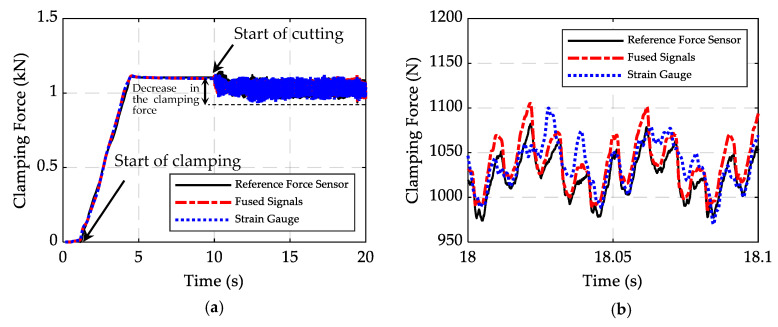
Comparing the measured forces: (**a**) from the start of clamping followed by cutting; (**b**) at the cutting section.

Figure 21 shows the signal of the *X*-axis strain gauge and the fused signals for a clamping test, followed by a cutting test with the same cutting parameters mentioned in Table 4. To evaluate the performance of the setup, the reference force sensor was also used for the measurement of forces. Note that different time constants and capacitors are required to convert and amplify the signals of piezoelectric sensors in quasi-static and dynamic tests; otherwise, the measurements will not be accurate and might include saturation during the clamping test or drift of the signal during the cutting test. Thus, in this experiment, the reference signal was measured in two steps. First, the clamping force was measured using a long time constant and a high capacitor. Then, a short time constant and a lower capacitor were used to capture the dynamic forces, and the final DC value of the clamping step was added to the signal to find the total clamping force. Based on the observations from Figure 21, the results of the strain gauge show large discrepancies with the reference signal at the cutting section, although as expected the signals are very similar for the clamping section. However, the discrepancies between fused results and the reference signal are much less through the whole process, with an average error of 19% for the cutting section (note that to calculate the errors, the DC value of the clamping section was subtracted from the data for the cutting section; otherwise the errors would be less than 1%). This proves that the proposed method can be used for accurate measurement of the total clamping force.

The results of the clamping force measurement in Figure 21 show that the machining forces caused about a 15% decrease in the clamping force. This reduction is a function of the applied static load and the intensity of the cutting forces, as well as the machining conditions. If insufficient clamping force is applied for workpiece holding, this effect will be significant and can cause workpiece slippage during the machining operations. Events such as sliding, lift-off, and chatter, as well as the time of their occurrence, can also be identified through the evaluation of the sensed information. Moreover, the applied static load and the cutting forces can be measured separately using the signal of strain gauges and PZT sensors, respectively. One of the advantages of the vise-based force measurement system is that a variety of workpieces can be easily mounted. Additionally, the structure of the setup consists of flat steel plates requiring insignificant machining, which contributes to the low manufacturing cost.

There are some limitations regarding the developed setup. The vise with the embedded sensors in this research has limitations related to the frequency bandwidth, which degrades the performance of the embedded sensors. The results of the FRF tests reveal that the frequency bandwidth of the setup is about 500 Hz; therefore, if the cutting test is conducted at higher spindle speeds, the measured forces can be distorted. To compensate for the dynamics of the system in order to achieve higher frequency bandwidths, the expanded Kalman filter [40] can be used. Moreover, in all the tests reported in this paper, the workpiece was clamped in the vise at the same height. Thus, if the workpiece is clamped higher or lower than that, new calibration tests are required to get more accurate results. Furthermore, the thermal effects on the response of the PZT piezoelectric sensors and strain gauges have not been considered in this paper.

## 5. Conclusions

The measurement of cutting and clamping forces is critically important to monitor and optimize machining operations. This paper presents a novel, cost-effective force sensor system for the simultaneous measurement of cutting and clamping forces in milling operations. To achieve this goal, the setup was developed in the form of a vise with PZT piezoelectric sensors and strain-gauge-embedded jaws. Since the deterioration of voltages, charge leakage, and drifts is one of the problems with piezoelectric sensors in the presence of static forces, the piezoelectric sensors were predominantly used to measure the dynamic forces. The piezoelectric properties of the developed sensors were measured and compared with the commercial ones. The PZT sensors were embedded on a layered jaw to measure the dynamic forces in X, Y, and Z directions, while strain gauges were attached on a cross-shaped groove to predominantly measure the static forces. The locations of strain gauges were carefully chosen to limit crosstalk among the static forces. The PZT sensors and strain gauges were calibrated by modal, static, and cutting tests.

The performance of the setup was evaluated by conducting actual clamping and cutting tests. The results show that the strain gauges are more suitable for the measurement of static and low-frequency bandwidth operations, while the PZT sensors can measure the dynamic cutting forces more accurately. Thus, the signals were fused to find the total clamping forces more accurately. Using this method, forces with low frequencies are mainly measured by the strain gauges, and PZT sensors are used to measure forces with high frequencies. The results confirm the performance of this setup in measuring the cutting and clamping forces. However, the setup has some limitations related to the bandwidth, and the system dynamics are dependent on the workpiece dimensions and material. Thus, further studies should be conducted to overcome these limitations.

## Figures and Tables

**Table 1:** The Chemical Composition of The Lead Zirconate Titanate (PZT) Powder.

Constituents	Pb_3_O_4_	TiO_2_	ZrO_2_	Ni and Nb
Weight fraction (wt%)	70.40	9.14	9.26	11.20

**Table 2:** Crosstalk Results from Finite Element (FE) Simulations.

Direction of the Load	Crosstalk		
	*X*-Axis Strain Gauge	*Y*-Axis Strain Gauge	*Z*-Axis Strain Gauge
X axis	-	41%	−17%
Y axis	−23%	-	<1%
Z axis	21%	<1%	-

**Table 3:** Sensitivity Factors of the Strain Gauges and PZT Piezoelectric Sensors.

Strain Gauges	Sensitivity (µε/N)	Piezoelectric Sensors	Sensitivity (pC/N)
*X*-axis	0.013	*X*-axis left	71
		*X*-axis right	137
*Y*-axis	0.020	*Y*-axis left	125
		*Y*-axis right	125
*Z*-axis	0.003	*Z*-axis left	67
		*Z*-axis right	100

**Table 4:** Cutting Parameters of the Milling Test.

Cutting Parameter	Value
Tool Diameter (mm)	12.7
Tool Length (mm)	3.7
Number of Flutes	4
Tool Material	Tungsten carbide
Depth of Cut (mm)	1
Feed Rate (mm/s)	10
Spindle Speed (rpm)	1500
Workpiece Dimensions L × W × H (mm)	120 × 50 × 50
Workpiece Material	AL 6061 T6511
